# Remodeling of the Posterior Cerebral Artery P1-Segment after Pipeline Flow Diverter Treatment of Posterior Communicating Artery Aneurysms

**DOI:** 10.3390/neurolint13020020

**Published:** 2021-05-07

**Authors:** Miguel S. Litao, Jan-Karl Burkhardt, Omar Tanweer, Eytan Raz, Paul Huang, Tibor Becske, Maksim Shapiro, Howard Riina, Peter K. Nelson

**Affiliations:** 1 Department of Neurology, NYU Langone Medical Center, New York, NY 10016, USA; 2 Department of Neurosurgery, University of Pennsylvania, Philadelphia, PA 19104, USA; Jan.Burkhardt@pennmedicine.upenn.edu; 3 Department of Neurosurgery, Baylor College of Medicine, Houston, TX 77030, USA; omar.tanweer@bcm.edu (O.T.); Paul.Huang@nyulangone.org (P.H.); Maksim.Shapiro@nyulangone.org (M.S.); Howard.Riina@nyulangone.org (H.R.); 4 Department of Radiology, Section of Interventional Neuroradiology, NYU Langone Medical Center, New York, NY 10016, USA; eytan.raz@nyulangone.org (E.R.); Peter.Nelson@nyulangone.org (P.K.N.); 5 Department of Neurosurgery, NYU Langone Medical Center, New York, NY 10016, USA; tiborb98@yahoo.com; 6 Department of Neurology, UNC Health, Raleigh, NC 27514, USA

**Keywords:** pipeline embolization, flow diversion, vessel remodeling, posterior communicating artery aneurysms

## Abstract

Introduction: Flow diverters such as the pipeline embolization device (PED) cause hemodynamic changes of the treated vessel segment. In posterior communicating artery (PcomA), aneurysms’ unique anatomic consideration have to be taken in account due to the connection between the anterior and posterior circulation. We hypothesize that in conjunction with PcomA remodeling, there will also be remodeling of the ipsilateral P1 segment of the posterior cerebral artery (PCA) after PED treatment for PcomA aneurysms. Methods: We retrospectively collected radiological as well as clinical data of PcomA aneurysm patients treated with PED including PcomA and P1 vessel diameters before and after treatment as well as patient and aneurysm characteristics. Results: Overall, 14 PcomA aneurysm patients were included for analysis and PED treatment was performed without complications in all patients. In 10 out of 14 patients (71%), a decrease in PcomA diameter was observed and there was a significant mean decrease of 0.78 mm in PcomA diameter on angiographic last follow-up (LFU) (*p* = 0.003). In the same patient population (10 out of 14 patients), there was meanwhile a significant mean increase of 0.43 mm in the ipsilateral P1 segment diameter observed (*p* = 0.015). These vessel remodeling effects were in direct correlation with aneurysm occlusion since all of these patients showed aneurysm occlusion at LFU while 29% showed only partial occlusion without vessel remodeling effects. A decrease in PcomA diameter was directly associated with aneurysm occlusion (*p* = 0.042). There were no neurologic complications on LFU. Conclusion: In the treatment of PcomA aneurysms with PED, the P1 segment of the PCA increases in diameter while the PcomA diameter decreases. Our results suggest that this remodeling effect is associated with aneurysm occlusion and decrease of PcomA is hemodynamically compensated for by an increase in the ipsilateral P1 diameter.

## 1. Introduction

Treatment of intracranial aneurysms with flow diverters such as the pipeline embolization device (PED) has been shown to be safe and effective for treatment of intracranial internal carotid artery aneurysms [1,2,3]. Its use for posterior communicating artery aneurysms (PcomA) is discussed controversial in the literature due to relatively high aneurysm recurrent rates as well as potential ischemic complications [4,5,6,7,8,9,10]. The anatomic variety of the PcomA and its relationship to the ipsilateral P1 segment of the posterior cerebral artery (PCA) further complicates standardized selection for PED treatment [5,8]. De Carvalho et al. investigated before the relationship of the P1/PcomA ratio with hemodynamic changes in the PcomA if a flow diverter stent is placed across the origin of the PcomA [8]. A P1/PcomA ratio of >1 was more likely to have flow changes in the PcomA compared to those with P1/PcomA ratio of <1. Besides the PcomA itself, there is no literature to our knowledge on vessel remodeling of the proximal PCA in the setting of PED treatment for PcomA aneurysms. We hypothesize that the resultant anatomic changes in the PcomA that accompany flow diverter origin coverage may lead also to anatomic changes in the P1 segment given the putative hemodynamic balance between the two vessels to preserve sufficient blood supply in the posterior circulation.

## 2. Materials and Methods

### 2.1. Patients and Data Collection

We retrospectively collected radiological and clinical data on all patients with PcomA aneurysms that were treated with PED over a 10-year time period from January 2008 to June 2018. Radiological parameters included the diameter of the ipsilateral PcomA and its ipsilateral P1 segment of the PCA before treatment and on the last angiographic follow-up as well as aneurysm size and angiographic treatment result. Clinical data included patient age, sex, and clinical co-morbidities. This study was approved by the Institutional Review Board (IRB).

### 2.2. PED Procedure

All patients received peri-operative dual antiplatelet therapy with aspirin and plavix 5–7 days prior to PED procedure and all patients received preoperative P2Y12 testing for antiplatelet response. Intra-procedural treatment with abciximab and/or heparin was depending on the preoperative P2Y12 result and plavix non-responder were switched to ticagrelor after the PED procedure. Pipeline deployment was carried out using a tri-axial system composed of a 6F guide catheter, a 0.058 inch inner diameter intermediate catheter, and a 0.027 inch inner diameter microcatheter. The number of pipeline stents was based on aneurysm size and aneurysm characteristics. Dual antiplatelet therapy was continued for 6 months until the first catheter angiogram follow-up and based on aneurysm occlusion reduced or continued.

### 2.3. Statistical Analysis

Data are presented as median with range for continuous variables and proportions for categorical variables. Independent t-test was used to assess for difference between groups while dependent t-test was used to assess for difference within the same group over time. Chi-square test was used to assess for categorical variables. Significance was assessed at *p* < 0.05. All analyses were performed using Statistical Package for the Social Sciences (SPSS) version 24 (IBM Corporation, Chicago, IL, USA).

## 3. Results

Overall, 15 PcomA aneurysms in 14 patients were treated with the PED and included in this analysis. One patient was excluded as a Neuroform stent (Stryker Neurovascular; Fremont, CA), was placed in a fetal PcomA before PED treatment, thereby potentially limiting or confounding vessel remodeling. The median patient age was 64 (range 38–82) with women comprising 12/14 (86%) of it. Median angiographic follow-up period was 9 months (range 2–63 months), with a median clinical follow-up period of 16.5 months (range 2–94 months).

Median aneurysm size was 5.68 mm (range 1.50 to 14.50 mm) and 10/14 (71%) patients presented unruptured while 4/14 (29%) patients presented previously with subarachnoid hemorrhage, albeit remotely with the most recent one being 9 weeks prior to treatment with the PED. Nine out of 14 (64%) patients had PED as stand alone treatment of the aneurysm, while 2/14 (14%) patients had concomitant coiling while another 2/14 (14%) patients had previous coiling. One patient (7%) was previously clipped. The mean number of stents used was 1.64 (range 1–4), with single PED in 7/14 (50%) patients, 2 PEDs in 6 (43%) patients, and 4 PEDs in 1 (7%) patient, respectively.

**Figure 1:**
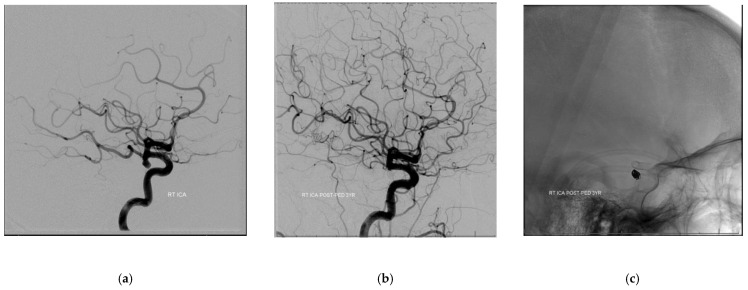

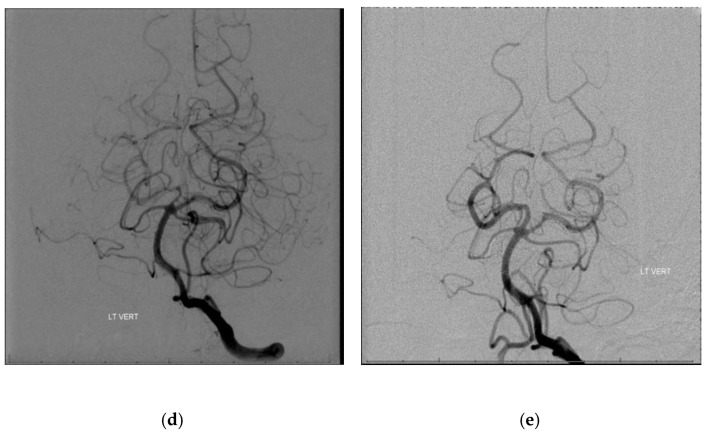
Illustrative case. (**a**–**c**) 5 mm posterior communicating artery aneurysm with a dominant posterior communicating artery treated with coils and one Pipeline stent showing persistent obliteration of aneurysm on 3 year follow-up as well as decrease in diameter of the dominant PcomA. (**d**,**e**) See increase in diameter of the ipsilateral (right) P1 segment of the posterior cerebral artery.

There was a significant mean decrease in the diameter of the PcomA on angiographic follow-up of 0.78 mm (*p* = 0.003) from 1.55 to 0.77 mm (SD = 0.21). Overall, 10/14 (71%) patients demonstrated a decrease in PcomA diameter while 3 (21%) patients showed no change and 1 (7%) patient showed an increase in diameter, respectively. In patients with a measured decrease in PcomA diameter, 4/10 (40%) patients showed no patency of the PcomA on angiographic follow-up. All patients with a P1/PcomA ratio of <1 (5/5, 100%) showed a PcomA diameter decrease compared to 5/9 (56%) patients with a P1/PcomA ratio >1 showing a PcomA decrease (*p* = 0.21), indicating that dominant PcomA tend to decrease in size as part of its remodeling. A decrease in PcomA diameter was associated with total aneurysm occlusion, showing a 90% (9/10) total occlusion rate (*p* = 0.042). Meanwhile, there was a significant mean increase in the diameter of the ipsilateral P1 segment of the PCA to the treated PcomA aneurysm on angiographic follow-up of 0.43 mm (*p* = 0.015) from 2.09 mm (SD = 0.43) to 1.66 mm (SD = 0.63). This difference is particular driven by patients with P1/PcomA ratio of <1, with a mean change of 0.86 mm versus patients with a P1/PcomA ratio of >1 with a mean change of 0.19 mm (*p* = 0.027). Most of the patients showed an increase in the P1 diameter (10/14, 71%) while 2 (14%) patients showed no changes and 2 (14%) other patients showed a decrease in the P1 diameter. Interestingly, all 4 patients with non-patent PcomAs on follow-up had ipsilateral P1 segment diameter increase at last follow-up (see Figure 1 for illustrative case).

Overall, 10/14 (71.4%) patients showed complete aneurysm occlusion on angiographic follow-up while 4 (28.6%) patients showed partial occlusion. There was no significant difference in aneurysm size between those partially occluded versus those with complete occlusion (7.69 mm vs. 5.54 mm, *p* = 0.30). There was also no difference in PED number used between those with partial versus complete aneurysm occlusion (1.75 vs. 1.60, *p* = 0.78).

Patients with stand alone PED aneurysm treatment showed a complete aneurysm occlusion on angiographic follow-up in 55.6% of the cases (5/9) while it was 100% (5/5) for patients with additional treatment methods (2 had prior coiling, 2 had concomitant coiling, and 1 had prior clipping) (*p* = 0.078).

**Figure 2:**
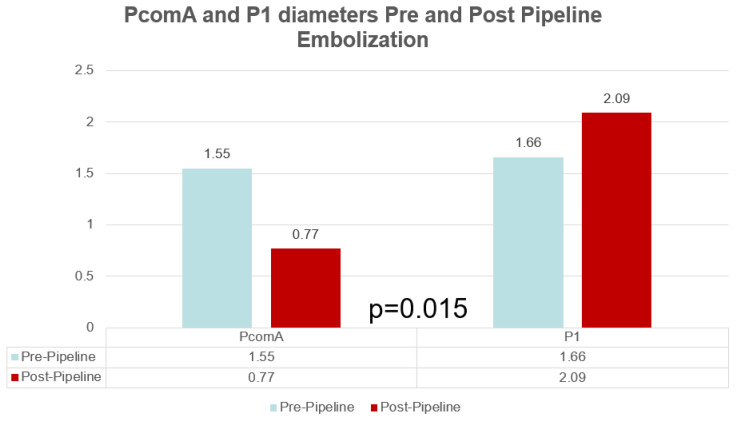
PcomA and P1 diameters pre and post pipeline embolization.

**Figure 3:**
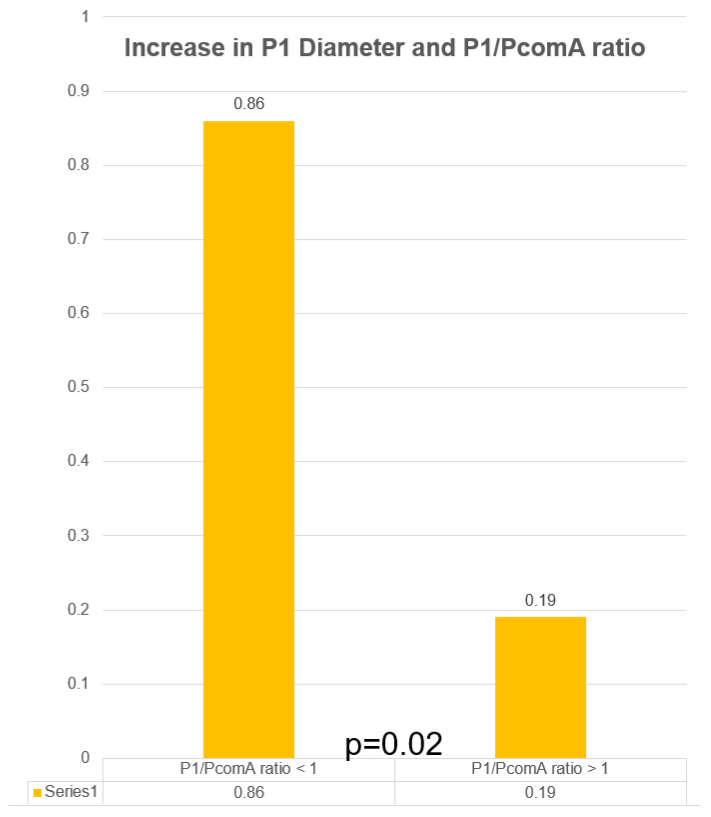
Change in P1 diameter in relation to P1/PcomA ratio.

There were no neurologic complications observed in the perioperative or follow-up period. One patient (7%) demonstrated in-stent stenosis with angiographic evidence of hemodynamic compromise with no associated neurologic deficits observed. There was no aneurysm rupture/re-rupture during the follow-up period (see Table 1, Figure 2 and Figure 3 for summary of data).

## 4. Discussion

In this study, we analyzed the vessel remodeling effect after PED treatment for PcomA aneurysms with focus on the PcomA and ipsilateral P1 segment of the PCA. We were able to show that in the treatment of PcomA aneurysms with PED, the P1 segment of the PCA increases in diameter while the PcomA diameter decreases. Our results suggest that this remodeling effect is associated with aneurysm occlusion and decrease of PcomA is hemodynamically compensated for by an increase in the ipsilateral P1 diameter.

Previous studies showed that the PcomA diameter decrease is likely as a consequence of PcomA origin coverage of the PED [5,7]. Another study also showed that the ratio between the PcomA and the P1 segment modulates the hemodynamic and anatomic response of the PcomA upon flow diversion coveratge. The novelty of our study is that concomitant with this decrease in PcomA diameter, an increase in the P1 diameter was observed, attesting to the relative hemodynamic balance between the two vessels/vascular segments.

One particular concern that has been investigated in small series before is that of treatment of PcomA aneurysms in PcomA that have a fetal configuration—which have included those with hypoplastic P1 segments [4,6]. There is concern that coverage of a fetal PcomA would result in ischemic deficits in the corresponding vascular territory as well as inadequate aneurysm treatment [4]. Our series includes 3 patients with a dominant posterior communicating artery with hypoplastic P1 segments (which will be defined as fetal PcomAs in other series) and all showed an increase in the P1 segment with a mean increase in P1 segment diameter of 1.1 mm with a mean concomitant decrease in PcomA diameter of 1.23 mm. It is important to note that none of the patients had neurologic deficits as a consequence of the stent attesting perhaps to the hemodynamic compensatory mechanism afforded by the P1 segment remodeling.

The aneurysm total occlusion rate in our series is 71% with the rest achieving partial occlusion, with a trend towards higher total occlusion rates in patients who had other treatment modalities in the past (coiling or clipping) or had concomitant coiling. This perhaps suggests that in given the flow demand through the PcomA that can potentially feed into the aneurysm, adjunct measures such as coiling be entertained if the purpose is to achieve total aneurysm occlusion sooner than later. It should be noted that no aneurysms ruptured or re-ruptured during the follow-up period.

The retrospective nature of this study limits the conclusions that can be derived from it. We think, however, that the concept of hemodynamic vessel remodeling of the PcomA and the ipsilateral P1 segments as a response to flow diverter treatment should be salient in one’s decision-making regarding treatment of these aneurysms.

## 5. Conclusions

In the treatment of posterior communicating artery aneurysms with Pipeline flow diversion, the P1 segment of the posterior cerebral artery remodels as the posterior communicating artery remodels. Our results suggest that a decrease in the PcomA diameter is associated with aneurysm occlusion and can be hemodynamically compensated for by an increase in the ipsilateral P1 diameter.

## Figures and Tables

**Table 1:** PcomA diameter and P1 diameter before and after PED treatment.

Patient	PcomA Diameter (mm)		P1 Diameter (mm)		Number of PED Stents	Treatment	Rupture Status (R = Rupture, U = Unruptured)	Aneurysm Occlusion (O = Occluded; P = Partially Occluded)
	Before	After	Before	After				
1	0.70	0	1.9	2.2	1	PED	U	O
2	2	0.8	0.8	2.2	1	PED/coil	U	O
3	1.60	2.30	1.9	2.3	2	PED	U	P
4	2.30	1.60	0.6	1	4	PED (previously coiled)	U	O
5	0.8	0.8	2	2	1	PED	U	P
6	1.9	1.9	2	3	2	PED	R (3 years before PED)	P
7	2.3	0.5	1.9	2	1	PED	U	O
8	1.6	0.82	1.8	2	2	PED	U	P
9	0	0	2.6	2.3	2	PED	R (9 weeks before PED)	O
10	0.9	0	1.7	1.9	1	PED/coil	U	O
11	1.43	0	2.21	2.1	1	PED (previously coiled)	R (8 months before PED)	O
12	1.4	1.30	1.8	1.8	1	PED	U	O
13	3	1.2	0.4	2	2	PED	U	P
14	2	0	1.6	2.4	2	PED (previously clipped)	R (3 years before PED)	O
